# Is there an association between periodontal disease and infertility? A systematic review

**DOI:** 10.4317/medoral.26831

**Published:** 2024-10-13

**Authors:** Cecilia Fabiana Márquez-Arrico, Francisco Javier Silvestre, Meylin Fernández-Reyes, Javier Silvestre-Rangil, Milagros Rocha

**Affiliations:** 1 University of Valencia, Stomatology Department, Valencia, Spain; 2 Service of Stomatology, University Hospital Doctor Peset, Valencia, Spain; 3 Service of Endocrinology, Foundation for the Promotion of Health and Biomedical Research in the Valencian Region (FISABIO), Valencia, Spain

## Abstract

**Background:**

Today, idiopathic infertility is becoming more frequent, affecting more than 186 million people in the world. The presence of comorbidities makes patient management complex, requiring individualized infertility treatment. Periodontal diseases could contribute negatively to the management of infertility, increasing inflammation, oxidative stress and insulin resistance, and contributing negatively to the development and progression of comorbidities associated with these two entities. The aim of this systematic review is to explore whether there is an association between periodontal diseases and male and female infertility and deepen into the possible mechanisms underlying this association.

**Material and Methods:**

The studies analyzed in this research included a total of 4871 patients (732 men and 4139 women), were original studies with high quality, mostly with a control group. Authors who have investigated idiopathic infertility suggest that infertility is associated with diseases that present low-grade chronic inflammation, oxidative stress and insulin resistance (such as obesity, type 2 diabetes and polycystic ovary syndrome), which are in turn related to periodontal diseases.

**Results:**

A higher prevalence of periodontal disease was found in patients with infertility compared with controls. Periodontal diseases could initially be mediated by a local and systemic proinflammatory environment, which favors a pro-oxidant state, leading to oxidative stress and, finally, irreversible destruction of the periodontal tissue. Insulin resistance, oxidative stress and inflammation are present in the pathologies associated with an increase in the prevalence and severity of periodontal diseases (such as obesity, type 2 diabetes and polycystic ovary syndrome). Therefore, IR, low-grade chronic inflammation and the oxidative stress could be the pathophysiological mechanisms linking idiopathic infertility and periodontal diseases.

**Conclusions:**

Studies suggest an association between infertility and periodontitis. Future researches are necessary to find causality factors. Studying the patient in a global and multidisciplinary way could help in the management and treatment of idiopathic infertility.

** Key words:**Infertility, periodontal diseases, insulin resistance, periodontitis, inflammation.

## Introduction

Infertility, according to the World Health Organization (WHO), is a problem characterized by the inability to achieve pregnancy despite having regular unprotected sex for one year (1). In 2010, it was estimated that 48.5 million couples worldwide were infertile, however, today; infertility is expected to be increasing, affecting more than 186 million people (8-12% of couples in reproductive age) and acts affecting the development of countries. A 25% of couples are affected in the Western world and a 14% in developing countries (2). The consequences of infertility can hinder population growth and could have a negative impact on the marital relationship, sexual satisfaction and social well-being (couples may experience anger, depression, anxiety, guilt, shame, altered self-esteem, feeling loss of control and incompetence, isolation) (2-4).

Infertility is a multifactorial condition for both partners and can be classified as primary or secondary. In women, primary infertility represents a woman who has never been diagnosed with a clinical pregnancy and meets the criteria for infertility. Secondary infertility refers to the inability to become pregnant or carry a baby to term after a previous successful pregnancy. Male infertility means a man is not able to start a pregnancy with his female partner (5).

Common risk indicators for infertility are environmental life style (sedentary lifestyle, poor diet, exposure to pollutants and radiation, alcoholism, drug addiction, among others) (6) and age (7). In addition, female infertility has been related to various systemic pathologies that generate chronic inflammatory conditions, including obesity or polycystic ovary syndrome (PCOS) (8), endometriosis, pelvic inflammation (3); as well as other indicators of risk of infertility could be, obstruction of Fallopian tubes, ovaries, or uterine problems. In the meanwhile obesity, diabetes type 2 (TD2) (9), hyperprolactinemia, hypogonadism, thyroid disorders and systemic infections, have been also linked to male infertility (10).

Recently, periodontal diseases have been comprehensively linked to male (11) and female infertility (3,8). Periodontal diseases are inflammatory pathologies of bacterial origin that causes a series of symptoms as gingival inflammation and bleeding of the gums, or eventually tooth loss. Moreover, these pathologies include two wide groups of pathological processes: gingivitis and periodontitis. Gingivitis is a reversible inflammatory lesion where there is no loss of the supporting tissues (bone, periodontal ligament and gingiva). Periodontitis is an irreversible infection that causes the resorption of the tooth-supporting tissues (12). One of the non-surgical treatments most used in patients with periodontitis is scaling and root planning (SRP) that it has been shown not only to ameliorate periodontal inflammation (reduction of bleeding on probing (BOP) and probing depth (PD), but also systemic inflammation by reducing hsCRP and TNF-α (13).

It has been shown that periodontal diseases are highly prevalent among infertile men and severe periodontitis was associated with further deterioration in sperm quality (14). On the other hand, men with periodontitis have a greater risk of developing concomitant erectile dysfunction, oligospermia, and asthenozoospermia (decreased percentage of motile spermatozoa) than healthy individuals. Taking these studies into account, a bidirectional association could be established between periodontal diseases and infertility. On the other hand, in females, periodontitis have been linked to adverse pregnancy outcomes, such us as low weight by gestational age, birthweight preterm birth, miscarriage or pregnancy loss, and pre-eclampsia (15).

Since many of the systemic metabolic disease causing infertility are related to low-grade chronic inflammation such as obesity and T2D (16) and PCOS (17), it is tempting to speculate that inflammation associated to periodontal diseases could be a causal factor of infertility showing a common aetiology link among them. Therefore, the aim of this systematic review is to explore whether there is an association between periodontal diseases and male and female infertility and deepen into the possible mechanisms underlying this association.

## Material and Methods

We carried out a systematic review following the criteria of the “Preferred Reporting Items for Systematic Reviews and Meta-Analyses” (PRISMA) guideline (18). The following question was posed: "Is there an association between periodontal diseases and infertility?” To structure our research question, we used the evidence-based PICO model (P: Patients; I: Interventions; C: Comparison; O: Outcomes) (19).

(P) Participants: infertile male and female. (I) Interventions: evaluation of periodontal status in patients with infertility. (C) Comparison: fertile male and female, of similar age and weight, who have undergone medical and periodontal evaluations. (O) Outcomes: periodontal clinical parameters in patients with and without infertility.

- Selection of articles

**Figure 1:**
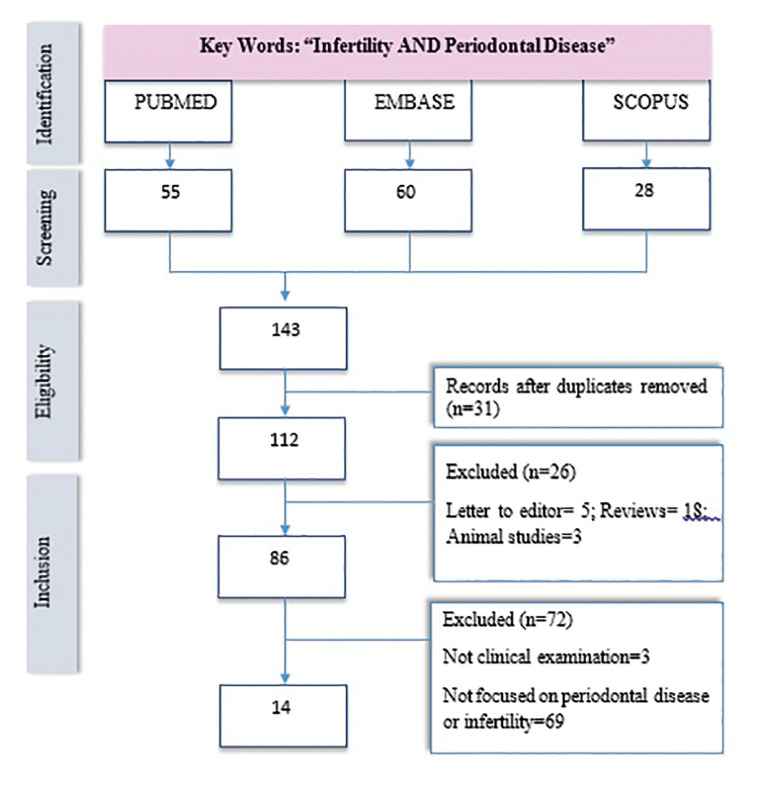
Flow chart.

The following keywords were used in three databases, Pubmed (National Library of Medicine, Washington, DC, USA), EMBASE and Scopus (Elsevier B.V): “Infertility” AND “Periodontal Disease” (Fig. 1).

We reviewed all original articles, cross-sectional, experimental, case-control and cohorts articles published until May 2024. Only articles in humans were taken into account, discarding studies in animals and review articles.

- Quality of articles

A quality analysis of the studies was carried out using the Newcastle-Ottawa scale (20) for case-control study and Jadad scale (21) for randomized controlled clinical trial. The Newcastle-Ottawa scale evaluates the methodological quality taking into account 3 criteria: selection, comparability and exposure assigning 3 points per section for a total of 9 points (Table 1).

The Jadad scale (21) evaluates the quality of randomized controlled clinical trials using parameters such as randomization of participants, and other methodology´s using with five question . Each question can be answered with yes/no, assigning 1 point for each affirmative answer. A maximum of 5 points can be obtained (Table 2). Both scales are used to carried out the risk of bias assessment.

- Infertility diagnosis

All the studies included in this review have diagnosed infertility as that situation in which the male or female patient has not been able to conceive a pregnancy after one year without any apparent medical explanation.

- Periodontal disease diagnosis

To diagnose the different types of periodontal diseases, the following periodontal indices were used: BOP; PD; loss of clinical attachment level (CAL); and oral hygiene indices such us plaque index (PI) (Table 3).

## Results

A total of 143 articles were found. After reading the abstract (screening) and analysis of the quality articles (eligibility), only 14 studies (Fig. 1) (3,4,7,14,22-31) answered our PICO question (inclusion), following the PRISMA protocol (18). Six studies were based on male infertility (14,22,24,26,27,31) and eight on female infertility. The studies analyzed in this research included a total of 4871 patients (732 men and 4139 women), (3,4,7,23,25,28-30) (Table 3). We analyse six case control studies (7,14,22,25,30,31), tree cross sectional studies (24,26,27), one observational study (29), two pilot study (3,4) and two randomized clinical trial (23,28).

- Periodontal parameters used

The parameters most commonly used in the periodontal evaluation were BOP (3,7,14,26-29,31), CAL (3,7,14,26,27,29,30) and PD (4,7,14,22,27,31). Oral hygiene and plaque level were assessed using the PI (3,4,7,26-28), oral hygiene index (29,30) and percentage of calculus (22,31). Some authors used other variables in the periodontal diagnosis such us bleeding index (4), sulcus bleeding index (30), number of missing teeth (22,27), periodontal inflamed surface (PISA) (3,7) periodontal epithelial surface area (PESA) (3,7) and only one study (27) carried out and x-ray examination to confirm or discard alveolar bone loss (Table 3).

- Periodontal parameters and female infertility

Regarding female infertility, some authors affirm that there is an association between periodontitis and infertility (3,7,28,30). Greater gingival inflammation - measured as the gingival inflammation index (a visual score), BOP, bleeding index and sulcus bleeding index - has been found in infertile women compared to control patients with a similar PI (4,7,28-30) (Table 3).

Lalasa *et al*., (30) and Machado *et al*., (3) found a higher CAL in women who came from assisted reproductive techniques compared with the control group. It can be observed how female patients who present infertility, have an increase PD and higher CAL, compared with the control group with similar oral hygiene habits, age and similar sociocultural characteristics.

The first line of treatment in assisted reproduction techniques is drug-induced ovulation. In this sense, the effect of ovulation-inducing drugs (clomiphene citrate and letrozole) , that raise levels of follicle-stimulating hormone (FSH) (28), on periodontal tissues showed that higher FSH and estrogen levels were associated with higher rates of gingival bleeding (28-30) (Table 3).

Yildiz *et al*., (7) reported a worsened periodontal profile (BOP, CAL,GI, PD, PI, PISA and PESA) in infertile women compared with fertile ones despite bacterial plaque (determined as PI) remains unaltered between groups. Only one investigation raised that the presence of different degree of gingival inflammation did not have an effect on the clinical fertility parameters (4). Hart *et al*., (23) shows how of the 146 women who took more than 12 months to conceive, 34% had periodontitis. It also establishes an OR of 2.88 for women exposed to periodontitis, taking more than a year to conceive. Similarly, Šumilin *et al*., (25), in their recent study found more inflammatory markers in the saliva of women with infertility than fertile women, but without finding significant differences in periodontal clinical results in favor of infertility.

- Periodontal parameters and male infertility

Gingival inflammation, determined mainly as BOP, was found to a greater extent in male infertile patients in all studies compared to controls (14,22,26,27,31). Additionally, there was a positive association between poor periodontal parameters (higher CAL, BOP and GI) and sperm count and submotility (14,22,26,27,31) (Table 3).

As a whole these worsened periodontal parameters in patients with infertility exhibited an increased prevalence of gingivitis and periodontitis , reaching 40% and 75%, respectively (26,27).

Tao *et al*., (14) and Pásztor *et al*., (22) studied how altered periodontal parameters could influence sperm quality and motility, finding a significant association between poor sperm quality and less sperm motility with moderate (CAL≥3mm or PD≥5mm) and severe periodontitis (CAL≥6mm or PD≥5mm).

Chidambar *et al*., (26) examined a group of 85 infertile male patients and carried out a comprehensive periodontal examination. These results were in line with Práger *et al*., (31) who analysed 199 patients, being 99 infertile patients and 100 controls. The BOP was found to be significantly higher in the infertility group (with the diagnosis of decreased sperm count - asthenozoospermia and oligospermia).

Nwhator *et al*.,(24) indicate that high plaque indices are significantly associated with low sperm count, being an easily modifiable risk factor that denotes the importance of good oral hygiene habits for our health. In the same way, patients with periodontal pockets are also associated with low sperm counts (24).

One of the most important indicators in periodontal disease is CAL, as it indicates the irreversibility of bone loss. An increase in CAL has been related to a decrease in sperm motility, a lower ejaculation volume and changes in morphology (26,27) (Table 3).

## Discussion

Up to now, only a few researchers have evaluated the association between infertility and periodontal diseases (3,4,7,14,22-31). One of the reasons that may justify this circumstance could be the multifactorial nature of both entities (1,10-12). Female and male infertility has been related to many systemic inflammatory diseases such as T2D (9,16), obesity (16,23,32), PCOS (3,8) or nutritional deficiencies and toxic habits such as tobacco, drugs, excessive alcohol consumption, among others (6). The study by Nwhator *et al*., (24) shows us how patients poor of oral hygiene and periodontal pockets are those with lower sperm counts. In addition, tobacco was associated with the presence of greater accumulations of tartar, being a risk factor associated with infertility.

Considering female infertility, Vasudevan *et al* (28) were the first to report an association between women infertility and periodontal disease, specifically gingivitis, showing how the administration of hormones that induce ovulation (such as chlomiphene citrate) is related to the increased incidence of gingivitis. In addition, the alteration of periodontal parameters has been related to poor outcomes in fertility treatments, suggesting that inflammation and infection at the level of the periodontium induce the release of inflammatory markers into the bloodstream, affecting implantation, adhesion and embryonic invasion (4). On its behalf, Machado *et al*. found a higher prevalence of periodontits in infertile women when compared with fertile ones. In order to identify causal factors, they took into consideration variables that influence the presence of periodontal pathologies, such as tobacco, oral hygiene or medication, reporting no differences between presence or absence of infertility. In fact, half of cases presented some type of periodontal disease (3). In this line, Yildiz *et al* (7) also found a significant association between infertility and the presence of higher PD and BOP that was independent of the oral hygiene habits - determined as PI and calculus index -. This fact suggests a causal effect between infertility and periodontal diseases and encourages researchers to deepen into underlying mediators that could be involved in such association. However, the studies in this review are focused on the clinical analysis and the patient's medical history, disregarding biochemical and metabolic parameters such as insulin, oxidative stress or inflammatory mediators.

Insulin is the major hormone involved in the cross-link between reproduction and metabolism. On one side, insulin modulates the gonadotrophin-releasing hormone (GnRH) receptors and regulates luteinizing hormone (LH) and FSH secretion that are essential to preserve the maturity of the gametes (oocytes and sperm) (16,33). Apart from that, insulin plays important role, as in glucose homeostasis, cell growth, and metabolism, and impaired insulin signalling and IR often leads to development of metabolic diseases such as obesity, T2D and classic phenotype of PCOS, all of them associated with infertility.

It has been demonstrated that obesity (34), T2D (9,16,35) and PCOS (3,8) are associated with a state of chronic low-grade inflammation, mediated by release of several inflammatory mediators from adipocytes and immune cells - such as tumor necrosis factor (TNF)-α, interleukin (IL)-6 and IL-1 - which have been involved in the development of IR and inflammatory response during periodontal disease (34,36).

Regarding obesity and IR, both have been linked to the increased prevalence of idiopathic infertility (16,32). Considering female infertility, hyperinsulinemia has been shown to contribute to hyperandrogenism and anovulation by mechanisms involving ovarian and adrenal androgen synthesis, and synergistically with LH, promoting the growth and formation of ovarian cysts by making them hyperresponsive to growth hormone, without allowing the selection and maturity of a single follicle and the corresponding ovulation (37). Therefore, obesity is associated with PCOS through underlying mechanisms involving IR.

Similarly, in obese men there is an alteration of the hypothalamic-pituitary-gonadal axis that gives rise to hyperestrogenic hypogonadism. At the same time, adipokines from adipose tissue could cause low testosterone levels (as occurs with the negative feedback of leptin) and increased inflammation and thus contribute to infertility (32). Strikingly, IR has been also linked to the increased prevalence of periodontal diseases (34). In fact, many of the pathologies associated with IR (such as obesity, diabetes and PCOS (13,17,34,38) have been related to periodontal diseases,. In this regard, obesity has also related to periodontal diseases, specifically periodontitis (38). It is worth noting that our group established the association between IR and periodontitis in an obese population, both male and female patients. Thus, patients who presented IR, showed poor periodontal parameters, with large PD and higher rates of BOP (34).

As we have already mentioned, PCOS is related to female infertility through the anovulation that it can present. Furthermore, a recent systematic review confirmed that PCOS patients are more vulnerable to develop periodontal diseases such as gingivitis and periodontitis. This altered periodontal response in PCOS was associated with a proinflammatory status that seemed to increase susceptibility to periodontal disease (17).

At cellular level, activation of inflammatory pathways favours an imbalance in the redox balance that promote an excessive production of free radicals that antioxidant defence systems cannot counterbalance leading to oxidative stress (39). This circumstance triggers oxidative damage in different macromolecules, such as proteins, lipids and DNA which negatively affect cell functionality. In accordance with this, infertile men reported sperm DNA fragmentation, increased ROS and systemic inflammation (32). Previous studies have reported an association between male infertility and periodontal status (24,29). Chidambar *et al*., (26) reported a positive association between periodontitis and diminished sperm counts and periodontal parameters and sperm submotility. Similarly, Pásztor *et al* (31) reported that PD ≥4 mm was more frequent in men with a sperm abnormality than in the control group. Both studies presented idiopathic infertility which suggests the possible involvement of exacerbate inflammatory response or oxidative stress as underlying molecular mechanisms which may be mediated the association between infertility and periodontal disease. This inflammatory response was reflected by salivary metalloproteinase markers in the Šumilin *et al*., study (25) in those patients with a low sperm count.

Taking in mind that glycaemia and insulin influences the immunological status it is reasonable to assume that T2D patients are more vulnerable to the aggression of periodontopathogenic microflora (13,36) and more susceptible to develop periodontal diseases, especially periodontitis (36) Furthermore, diabetic patients with poor glycaemic control have reported an impaired healing capacity as consequence of impaired immunological response that could explain poor periodontal outcomes after both surgical and non-surgical treatment (13,34). Therefore, an effective periodontal treatment can contribute to control patients' glycaemia (13).

Within the limitations of this study we can mention the diversity in the methodology used by the different authors. However, the diagnostic criteria for infertility and periodontal disease have been the same in practically all the studies, giving homogeneity to the results compared in this systematic review.

## Conclusions

- Implications for clinical practice

To conclude, in this systematic review, we have focused on the bidirectional association between infertility and periodontal diseases. The presence of comorbidities such as IR characteristic of obese, PCOS and TD2 patients, trigger the release of proinflammatory cytokines such as TNFα that could initially be mediated by a local and systemic proinflammatory environment, which favours a pro-oxidant state, leading to oxidative stress and, finally, irreversible destruction of the periodontal tissue, contributing to pathophysiological mechanisms linking idiopathic infertility and periodontal disease.

Addressing the patient’s evaluation in a global and multidisciplinary way could help in the management and treatment of idiopathic infertility. Thus, the prevention and treatment of periodontal diseases to reduce IR, inflammatory mediators and the oxidative stress could contribute to the control of systemic pathologies, and improve the general health status of the patient, providing benefits in the conception process.

- Implications for research

In this context, additional research is warranted in order to clarify the relationship between infertility and periodontal diseases.

## Figures and Tables

**Table 1:** Quality analysis of the studies using the Newcastle-Ottawa Scale for case-control study.

Authors	Selection	Comparability	Exposure	Total score/9
Chidambar *et al.*, 2019	***	***	***	9/9
Khalife *et al.*, 2019	***	*	***	7/9
Klinger *et al.*, 2011	***	***	***	9/9
Lalasa *et al.*, 2014	***	***	***	9/9
Machado *et al.*, 2020	***	***	***	9/9
Nwhator *et al.*, 2014	***	***	**	8/9
Pásztor *et al.*, 2016	***	**	***	8/9
Práger *et al.*, 2017	***	***	***	9/9
Smadi *et al.*, 2017	***	***	***	9/9
Šumilin *et al.*, 2022	**	**	***	7/9
Tao *et al.*, 2021	***	***	***	9/9
Yildiz *et al.*, 2020	***	***	***	9/9

Selection: the score in this section depends on the representativeness of the exposed cohort, ascertainment of exposure and demonstration that outcome of interest was not present at start of study. Comparability: comparability of cases and controls on the basis of the design or analysis. Exposure: ascertainment of exposure, with objective methods, such us surgical procedures or structured interview where blind to case/control status. Same method of ascertainment for cases and controls. Same response rate to treatment or procedure for both groups. *: 1 point of total score.

**Table 2:** Quality analysis of the studies using the Jadad scale for randomized controlled clinical trial.

Authors	Item 1	Item 2	Item 3	Item 4	Item 5	Total Score
Hart *et al.*, 2012	1	1	0	0	0	2/5
Vasudevan *et al.*, 2013	1	1	0	0	0	2/5

1. Was the study described as randomised?; 2. Was the method used to generate sequence of randomisation described and appropriate?; 3. Was the study described as double blind?; 4. Was the method of double blinding described and appropriate?; 5. Was there a description of withdrawals and dropouts?. *:1 point of total score.

**Table 3:** General characteristics and main findings of total studies included in the systematic review.

Authors and Journal	Patients andStudy Design	Mean age Size sample	Periodontal Parameters	Results and Conclusions
Chidambar *et al.*, 2019 J Hum Reprod Sci	Male patients Cross sectional	21-45 years old n=85 NS= 23% OS=43% SM=76%	Nº of BOP CAL GI PI	A 24.7% of cases have gingivitis (GI between 1-4), and 75.8% of cases presented periodontitis (presence of CAL). Periodontitis was associated with diminished sperm counts. There was a positive association between poor periodontal parameters (higher CAL, BOP and GI) and sperm submotility. PI was associated with a higher CAL, BOP and sperm morphology.
Hart *et al.*, 2012 Hum Reprod	Female patients Randomizec controlled trial	3416 spontaneous conceptions, including 1014 cases with periodontal disease (29.7%). Planned pregnancies accounted for 1956 of the 3416 pregnancies	PD>4mm Tobacco Tonsils pathologies Sinus patologies	For 146 women, time to conception was >12 months and periodontitis was more prevalent in this group (34.9% versus 25.7%, P = 0.015). The median time in women with periodontitis was 7.1 months [confidence interval (CI): 5.7-8.6] compared with 5.0 months (CI: 4.4-5.5, P = 0.019) in those without periodontitis. Periodontitis was present in 23.8% of Caucasian women and 41.4% of non-Caucasian women. Women with periodontal pathology were more likely to have a conception time greater than one year compared to periodontally healthy women [13.9% versus 6.2%, odds ratio (OR): 2.88 (CI: 1.62- 5.12), P < 0.001], age, BMI >25 and smoking were also predictors.
Khalife *et al.*, 2019 Int J Womens Health	Female patients Pilot study	22-37 years old n=28 17 patients had a positive pregnancy test (60%) with a total of 13 live births (46%) and 4 pregnancy losses (14%)	Bleeding Index GI PD PI	Mild gingivitis (GI 1 = mild inflammation - slight change in color and little change in texture) was found in 47.1% infertile woman, 8.8% moderate gingivitis (GI 2 = moderate inflammation - moderate glazing, redness), and 23.5% severe gingivitis (GI 3 = severe inflammation with edema, hypertrophy and bleeding on pressure). Bleeding index,PD and PI were not associated with poor in vitro fertilization outcomes.
Klinger *et al.*., 2011 J Clin Periodontol	Male patients Cross sectional	32,7 years old n=75 NS=37% OS=48% AS=15% SM=30% AM=13%	Number of missing teeth Mobility of teeth BOP CAL GI PD PI X-ray	A higher CAL was found in SM compared with NS. A 48% of infertile patients presented periodontitis (PD ≥4mm in two or more teeth) and a 40% gingivitis (BOP and GI ≥1 in two or more teeth). Patients with sperm SM presented more loss of bone (in X-ray examination) than NS patients. Missing an mobility teeth, PI, were similar y all patients.
Lalasa *et al.*, 2014 Indian J Dent Res	Female patients Case-control	25-35 years old n=180 Group I=60 Infertility women under treatment Group II= 60 infertility women not treated Group III=60 controls	Oral hygiene index simplified Sulcus bleeding index CAL GI	Group I had significantly higher GI and sulcus bleeding index as compared to Group II and controls (p< 0.05). Furthermore, women in Group I and Group II had statistically higher CAL as compared with the controls (p< 0.05). Oral Hygiene index were similar in all groups. The infertility women under treatment showed more loss of bone compared with controls.
Machado *et al.*, 2020 Int J Environ Res Public Health	Female patients Pilot case-control	34 years old n=36 Cases=18 Controls=18	BOP Calculus CAL PD PI PISA PESA	Cases- patients referred for fertility treatment- presented a higher CAL, PD and PESA compared with control group. BOP, calculus, PI and PISA do not show significant differences between groups. Periodontitis was defined as detectable interdental CAL at ≥ 2 non-adjacent teeth; or buccal ororal CAL ≥ 3 mm with PD > 3 mm detectable at ≥2 teeth, and the observed CAL was not attributed to non-periodontal causes. Cases presented a higher occurrence of periodontits than in the control group.
Nwhator *et al.*, 2014 J Contemp Dent Pract	Male patients Comparative-study	86 participants There were 55 subjects with subnormal counts (of which ten were found to be AS, 41 OS) and 25 NS	CPITN OHIS PD	There was a significant association between periodontitis and subnormal sperm counts [0.02599 (CI = 0.141089 - 2.03891)] between subjects and controls only in the age group of 33 to 38 years. There was also a slight association (p = 0.08219) between subnormal sperm counts and have periodontal pocket (Code 3) demonstrated from a sextant analysis of cumulative Code 0-3 scores between subjects and controls.
Pásztor *et al.*, 2016 J Oral Sci	Male patients Case-control	35 years old n=95 Cases=63(OS=57%; AS= 42%) Controls=32 (NS=100%=)	BOP% Missing teeth Calculus% PD≥4mm	A significant higher BOP and PD≥4mm was presented in AS group compared with NS. The OS group showed a higher mean of PD and calculus, compared with NS. There was no significant association between sperm abnormality and messing teeth.
Práger *et al.*, 2017 J Clinical Periodontology	Male patients Case-control	34 years old n=199 Controls=106 Cases =93 OS=27%AS=23% CS=16% OS+AS=32%	BOP PD PI Calculus %	A significant association between BOP and calculus was found in infertility groups (combined OS and AS). PI and PD didn't showed differences between groups.
Smadi *et al.*, 2017 Indian J Dent Res	Female patients Clinical observational	n=179	BOP CAL Oral hygiene index simplified,	A higher BOP and GI was associated with high estrogen levels. There were no significant differences between the group of women who undergone invitro treatment and get pregnant. It was significant association with pregnancy and GI. CAL Oral hygiene index didn't show significant associations.
Šumilin *et al.*, 2022	Female patients Case-control	n=100 50 cases 50 controls	BOP CAL PD PI PISA	Patients with infertility showed higher levels of MMP-8 but without an increase in periodontal clinical parameters in the infertility group.
Tao *et al.*., 2021 Oral Dis	Male patients Case-control	20-50 years old n=192 Cases=63 Controls=129	BOP CAL PD	A significant association was found between periodontitis and infertile patients. A 33% of cases presented moderate periodontitis (CAL≥3mm or PD≥5mm) and a 17.8% presented severe periodontitis (CAL≥6mm or PD≥5mm). BOP was associated with poorer sperm quality.
Vasudevan *et al.*, 2013 J Contemp Dent Pract	Female patients Randomized controlled clinical trial	27 years old n=100 Infertility woman using ovulation induction drugs Groups A=CC<3 cycles; B= CC>3 cycles, C= Letrozole, D= no drugs	BOP GI PI	In the ovulation drugs group (Letrozole and CC) was found a higher BOP and GI compared with no drugs group. PI didn't showed differences between groups. GI is risk factor for pregnancy and pregnancy outcomes, thus its control by scaling would play a main role in the successful outcome of the infertility treatment.
Yildiz *et al.*, 2021 J Periodontol	Female patients Case-control	21-39 years old n=100 Cases=50 Controls=50	BOP CAL GI PD PI PISA PESA	BOP, CAL, GI, PD, were higher in cases group compared with controls (p≤0.05). PI, PISA and PESA didn't show significant association.

Abbreviations: AS: asthenozoospermic; AM: abnormal morphology; BOP: bleeding on probing; CAL: clinical attachment level; CC: clomiphene citrate; CPITN: community periodontal index of treatment needs; CS: cryptozoospermic; GI: gingival index; HCG: human chorionic gonadotropin; MMP-8: matrix metalloproteinases 8; NS: normospermic; OHIS: oral hygiene index score; OS: oligospermic; PD: probing depth; PESA: periodontal epithelial surface area; PI: plaque index; PISA: periodontal inflamed surface area; SM: sub-motility.
